# Targeting interleukin-1 receptor-associated kinase 1 for human hepatocellular carcinoma

**DOI:** 10.1186/s13046-016-0413-0

**Published:** 2016-09-13

**Authors:** Ning Li, Jinhua Jiang, Jing Fu, Ting Yu, Bibo Wang, Wenhao Qin, An Xu, Mengchao Wu, Yao Chen, Hongyang Wang

**Affiliations:** 1 State Key Laboratory of Oncogenes and Related Genes, Shanghai Cancer Institute, Renji Hospital, Shanghai Jiao Tong University School of Medicine, Shanghai, People’s Republic of China; 2 International Cooperation Laboratory on Signal Transduction, Eastern Hepatobiliary Surgery Institute/Hospital, 225 Changhai Road, Shanghai, 200438 People’s Republic of China; 3 National Center for Liver Cancer, Shanghai, 201805 People’s Republic of China; 4 The First Clinical Medical College, Fujian Medical University, Fuzhou, 350001 Fujian China

**Keywords:** Hepatocellular carcinoma, IRAK1, Proliferation, Cell cycle, Subcutaneous tumor, Apoptosis

## Abstract

**Background:**

Interleukin-1 receptor associated kinase 1 (IRAK1), as a down-stream of toll-like receptor (TLR) signaling, plays important roles in series of malignancies. However, the role of IRAK1 in hepatocellular carcinoma (HCC) remains little known.

**Methods:**

In our study, reverse transcription-PCR (RT-PCR), Western Blot, and immunohistochemical staining were used to assess the mRNA and protein levels of IRAK1 in clinical samples and cell lines. Cell counting assay and flow cytometry were employed to analyze the effect of IRAK1 on cell cycle and apoptosis. Transwell assay was used to study the role of IRAK1 in cell migration. Moreover, subcutaneous xenograft tumor models predict the efficacy of targeting IRAK1 against HCC in vivo.

**Results:**

IRAK1 was over-expressed in HCC tissues and cell lines. Suppression of IRAK1 by small interference RNA (siRNA) or a pharmaceutical IRAK1/4 inhibitor impeded cell growth, induced apoptosis and lessened HCC xenograft tumor growth. Particularly, IRAK1/4 inhibitor treatment caused G1/S cell cycle arrest and apoptosis, confirming IRAK1 as a new therapeutic target for HCC.

**Conclusion:**

IRAK1 promotes cell proliferation and protects against apoptosis in HCC, and can be a novel target for HCC treatment.

## Background

Primary hepatocellular carcinoma (HCC), one of the most common malignant solid tumors, is a prototype of inflammation-related cancer due to a history of uncontrolled inflammation and cirrhosis, and most human HCC cases are related to inflammation and cirrhosis [1–5]. Previous reports suggested that the chronic damage and inflammation had closed relationship with carcinogenesis [6]. For example, interleukin-1β (IL-1β) is a critical inflammatory cytokine linking with chronic inflammation [7, 8]. The gender difference of liver cancer is the result of sex-associated differential production of IL-6, which relies on IL-1R/IRAK-1 signaling [9]. In addition, IL-1β/IRAK-1 signaling contributes to persistent expression of oncogene *Gankyrin*, and the later promotes HCC progression [8]. Aberrant IRAK1 expression has been shown in multiple tumors, such as myeloma, leukemia and several types of solid cancers. Accordingly, there has been much effort to target IRAK1 using pharmaceutical inhibitors [10–12].

IRAK1, the first protein to be discovered in IRAK family, is the main mediator of TLR/IL1R signal pathways. After IL1R/TLR binding, IRAK1 interacts with MyD88, which could be rapidly recruited to the receptor. The phosphorylation of IRAK1 is a multistep process and results in the activation of IRAK1. In this process, the threonine 209 (T209) is vital for IRAK1 kinase activity [13–15]. Phosphorylated IRAK1 could release from the receptor complex and bind to the E3 ubiquitin ligase and TRAF6. As the result, NF-kB signal pathway is activated [16–18]. In some malignant tumors, disordered inflammatory toll-like receptor (TLR) signaling is related to up-regulated NF-kB activity. The IRAK family members are mediators of TLR/IL1R signal pathways, and amounts of evidence implicate that these kinases including IRAK1 are vital cancer targets [19, 20]. IRAK1 is also related to the formation and development of series of myeloid malignancies or tumors, especially in myelodysplastic syndrome (MDS), acute myeloid leukaemia (AML) [21], melanoma [22], Lung Cancer [23, 24] and Breast cancer [25, 26]. However, the roles of IRAK1 in HCC have not been investigated by now.

Given that IL-1R/MyD88/IRAK signaling plays a critical role as a contributor to progression of HCC [8], the goal of our study was to gain a greater understanding of the significance of IRAK1 in the growth and survival of HCC. We found that HCC cells expressed elevated levels of IRAK1 mRNA as well as protein levels of total IRAK1. Inhibition of IRAK1 with siRNAs or a small molecular inhibitor retarded cell proliferation. At a mechanistic level, IRAK1 regulated G1/S phase of cell cycle and cell apoptosis. The importance of targeting IRAK1 in HCC was emphasized by demonstrating that treatment with IRAK1 siRNAs suppressed HCC tumor growth in xenograft model. Our study highlighted a critical of IRAK1 in HCC proliferation and suggested a pathophysiological role and clinical implication for patients with HCC.

## Methods

### Reagents and cell lines

All chemical reagents were purchased from Sigma-Aldrich (St Louis, MO, USA). The IRAK1/4 inhibitor, which selectively inhibits the kinase activity of IRAK1 at the IC50 of 0.75 mM and prevents TRAF6-mediated canonical NF-kB activation, has been developed for autoimmune disease and Myelodysplastic Syndrome [27–29]. Here, the IRAK1/4 inhibitor (I5409) dissolved in DMSO was used for the inhibition of p-IRAK1. Cisplatin (Cis) and epirubicin (ADM) were used for stimulating the cell apoptosis as previous report of our lab [8]. HEK293T, MHCC-LM3, SMMU-7721, HepG2, PLC/PRF/5 and PVTT cell lines were purchased from Cell Bank of Type Culture Collection of Chinese Academy of Sciences (Shanghai, China). Cells were grown in DMEM (Life Technologies, Carlsbad, CA, USA) which was supplemented with 10 % fetal bovine serum (FBS, Life Technologies), 100 U/ml penicillin and 100 μg/ml streptomycin, and at 37 °C with 5 % CO_2_.

### HCC samples and immunohistochemical straining

About 33 HCC samples were collected from Eastern Hepatobiliary Surgery Hospital (Shanghai, China) and the procedure of human sample collection was approved by the Ethical Committee of Eastern Hepatobiliary Surgery Hospital. Immunohistochemical staining was performed with total IRAK1 antibody (Santa Cruz, CA, USA) and Ki67 antibody (Santa Cruz) as reported previously [8].

### Lentivirus-mediated knockdown of IRAK1

Three si-IRAK1 lentiviruses were purchased from Hanbio (Shanghai, China). MHCC-LM3 and SMMU-7721 Cells were transfected with Si-IRAK1 lentivirus at multiplicity of infection (MOI) of 0.5 ~ 1 at the cell density of 10^5^/ml. At 48 h after transfection, puromycin was added to the cell medium for screening positive cells.

### Real-time PCR analysis

RNA was extracted with the Trizol reagent (Thermo Fisher Scientific, Hudson, NH, USA) and reverse transcription was carried out with SuperScript® IV Reverse Transcriptase (Thermo Fisher Scientific). Real-time PCR was performed with SYBR Green qPCR mix (Life Technologies) and Lightcycler 96 System (Roche Diagnostics, Rotkreuz, Switzerland) while 18 s cDNA was used as the reference.

### Cell migration analysis

Cell migration assay was performed with transwell chambers (Costar, Cambridge, MA) as the method of Binhui Xie et al. [30]. For SMMU-7721 cell lines, about 2 × 10^5^ cells were plated into the chamber with serum free-DMEM medium. Then, the chamber were placed into wells of the 12-well plate which was added with the medium containing 10 % FBS. After incubation for 24 h, the migrated cells were fixed with 4 % paraformaldehyde and stained with crystal violet for 10 min.

### CCK-8 and colony formation analysis

The CCK-8 analysis was carried out according to the instruction of Cell Counting Kit-8 (CCK-8, Dojindo, Tokyo, Japan). Briefly, IRAK1 inhibitor at concentrations of 0, 10, 20 μM were added into SMMU-7721, HepG2, PLC/PRF/5 and PVTT for 1–5 days. Then, 1/10 diluted CCK-8 solution with DMEM was added into the target wells of 96-well plate after discarding the culture medium, following the incubation for 1 h and measurement using a microplate reader (Bio-Rad Laboratories, Hercules, CA). Cell proliferation rates were calculated and normalization with the OD value of 1^st^ day.

Colony formation analysis was carried out in SMMU-7721 and HepG2 cell lines. The cells were plated into a 6-well plate (3 × 10^3^ cells per well) and incubated for 12 days. Cells were fixed with 4 % para-formaldehyde and stained with crystal violet.

### Cell cycle and apoptosis analysis

SMMU-7721 were plated into the 6-well plate and cultured in serum-free DMEM medium for 12 h in order to synchronize cells. Then the medium was changed into DMEM medium with 10 % FBS. After 24 h or 48 h, the cells were fixed with 70 % ethanol at 4 °C for more than 4 h and then washed by phosphate buffered saline (PBS). In order to remove RNA, RNase A (1 mg/ml of final concentration) was used to digest the fixed cells for 30 min. Then, the staining was carried out with 50 μg/ml propidium iodide (PI) in PBS-Triton X-100 for another 20 min at 4 °C. Finally, results were acquired by the flow cytometer (Life Technologies) analysis.

The cell apoptosis analysis was carried out with the previous method [31]. After treated with IRAK1/4 inhibitor (20 μM) for 24 h and 48 h, SMMU-7721 cells were stained with the Annexin V/PI kit (Life technology) according to the instruction. Cell apoptosis was detected and analyzed by the flow cytometer.

### Nucleocytoplasmic separation

The nucleocytoplasmic separation was carried out with the NE-PER nuclear and cytoplasmic extract Kit (Thermo Fisher Scientific). As the method described in this kit, SMMU-7721-SiIRAK1-1 and SMMU-7721-NC cell lines were cultured and harvested for extracting the cytoplasmic and nuclear proteins after stimulated by cisplatin (Cis) and epirubicin (ADM) for cell apoptosis. P65 was detected with Western Blot analysis while Histone H3 and GAPDH as the internal control.

### Western blot analysis

As the previously described methods [8], proteins (30 μg/lane) were isolated by SDS-PAGE electrophoresis and then transferred to NC membranes. Then, the membranes were blocked with 5 % milk in Tris–HCl buffer and 0.05 % Tween-20 (TBS/Tween) for 1 h and incubated with primary antibodies against total IRAK1(Santa Cruz), phosphorylated-IRAK-1 (Ser 209) (Cell Signaling Technology, Danvers, MA, USA), P65 (Cell Signaling Technology), GAPDH (Cell Signaling Technology), Histone H3 (Cell Signaling Technology) and β-actin (Cell Signaling Technology) overnight at 4 °C. Subsequently, the membrane was incubated with fluorescence-conjugated secondary antibody (Cell Signaling Technology) and scanned with Odyssey scanner (LI-COR Biosciences, Lincoln, Nebraska).

### In vivo subcutaneous tumor model

Five weeks old male nude mice were purchased from Shanghai Experimental Animal Center (Shanghai, China) and fed in Experimental Animal Center of Second Military Medical University (Shanghai, China). All animal experiments were carried out according to the directions of Second Military Medical University Animal Care Facility and the National Institutes of Health guidelines. SMMU-7721-SiIRAK1-1 and SMMU-7721-NC cell lines were cultured and subcutaneously injected at 5 × 10^6^ cells/nude mouse. About two months later, six nude mice were sacrificed for harvesting subcutaneous tumor. At last, the subcutaneous tumor samples were analyzed by immunohistochemical straining with the Ki67 antibody (Cell Signaling Technology).

For the IRAK1/4 inhibitor treatment, the IRAK1/4 inhibitor was dissolved in DMSO at the concentration of 5 mM, and further diluted in PBS. The SMMU-7721 cell line was implanted subcutaneously in mice and allowed to grow until the tumors reached a size of approximately 150 mm3. In vivo delivery of the IRAK1/4 inhibitor approach is adapted from previous reports of Yang. et al. [29] and Rhyasen GW. et al. [20], xenografted mice were randomized and injected i.p. with 2.12 mg/kg IRAK1/4 inhibitor four times per week for two weeks.

### Statistical analysis

All experiments were repeated for three times and the data analysis was performed with student *T*-test and two-way ANOVA analysis with GraphPad software. Tumor volumes from two groups in subcutaneous HCC models were analyzed by two-way ANOVA analysis. The data were presented as the mean ± SEM. *P* < 0.05 was considered statistically significant.

## Results

### IRAK1 was over-expressed in HCC tumor tissues

**Fig. 1:**
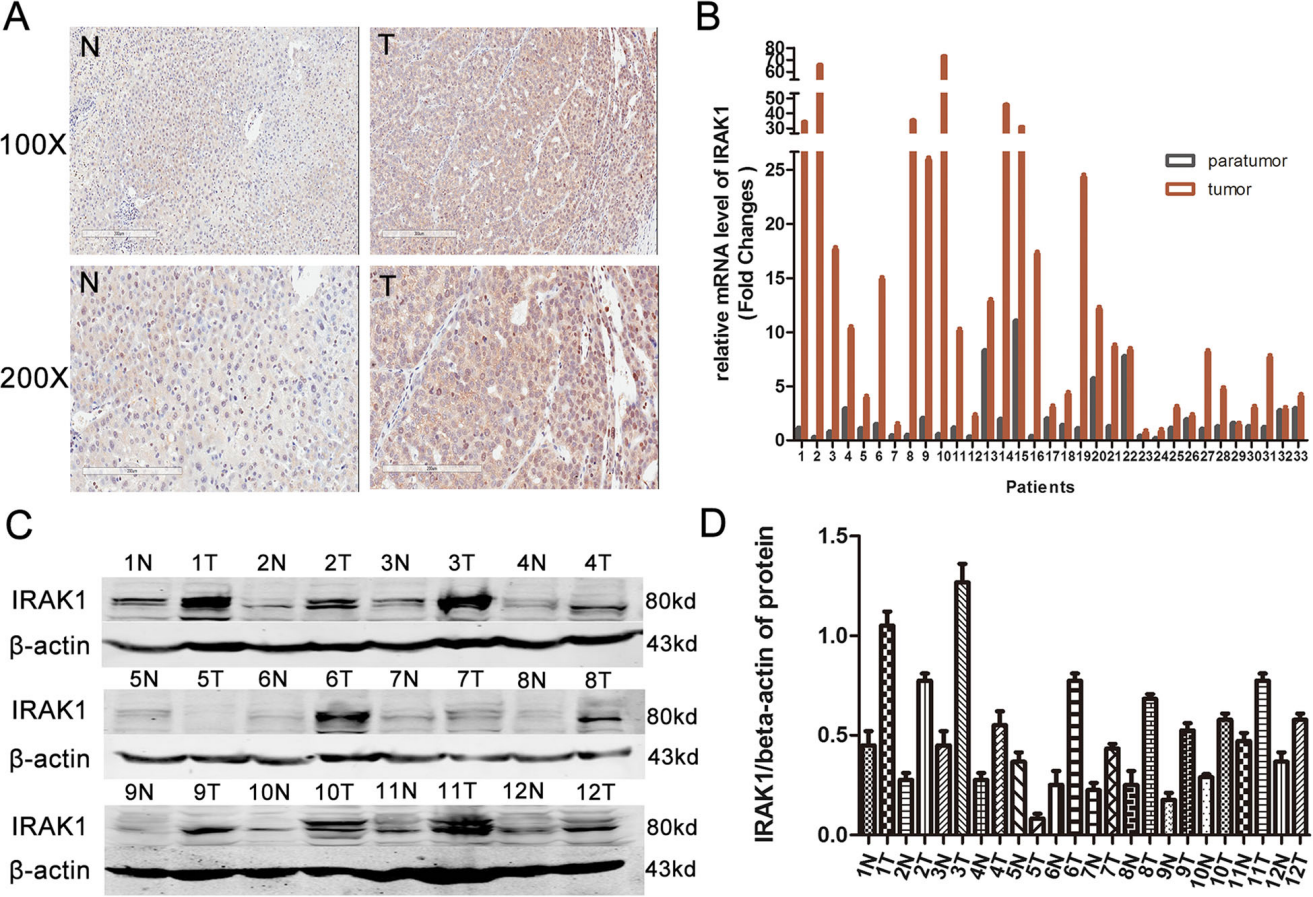
High expression of IRAK1 in HCC tissues (T) comparing with adjacent normal tissues (N). **a** IRAK1 expression was analyzed by IHC in 33 human liver tissues. Representative IRAK1 staining of de-paraffinized sections of adjacent normal liver (N) and HCC tumor (T) tissues. **b** RT-PCR analysis of IRAK1 mRNA in HCC tumor tissues. **c** Western Blot analysis of IRAK1 in HCC tumor tissues (T1-12) and adjacent liver tissues (N1-12). **d** The normalization expression of IRAK1/β-actin. Actin was used as a loading control

The mRNA and protein level of IRAK1 was investigated in 33 clinical HCC specimens. We found that IRAK1 was highly expressed in the HCC tissues comparing with adjacent normal tissues by immunohistochemical staining (Fig. 1a). IRAK1 was mostly located in the cytoplasm, while some existed in nucleus. Increased IRAK1 mRNA levels were comparable to those in non-malignant tissues (Fig. 1b). As is showed in Fig. 1c and d, increased protein levels of IRAK1 was detected in 12 HCC biopsies, suggesting that IRAK1 was over-expressed in most HCC specimens.

### Knockdown of IRAK1 reduced HCC cell growth

**Fig. 2:**
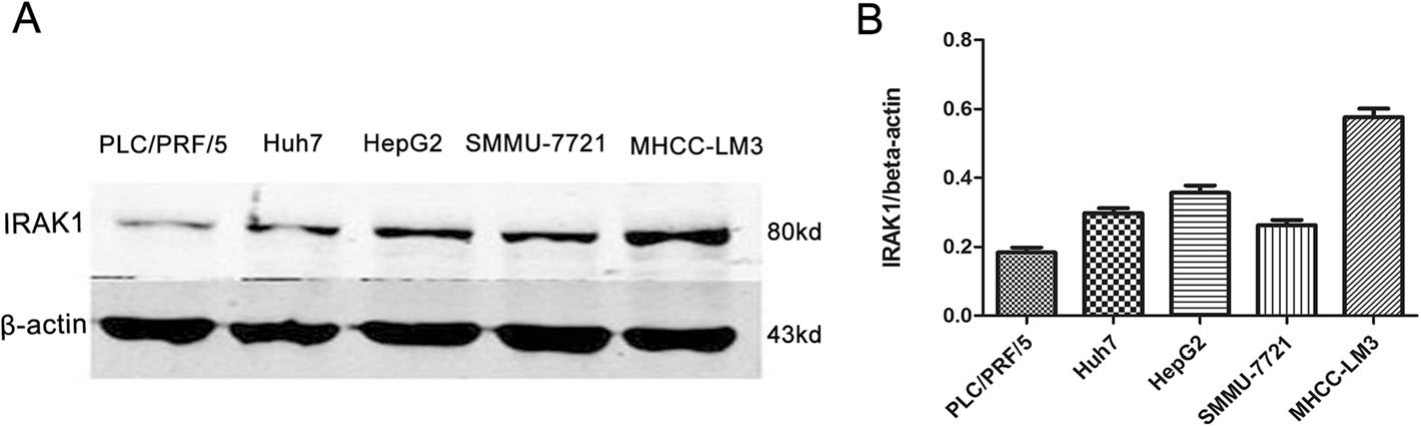
High expression of IRAK1 in different HCC Cell lines. **a** Western Blot analysis of IRAK1 in different HCC cell lines. **b** The normalization expression of IRAK1/β-actin

**Fig. 3:**
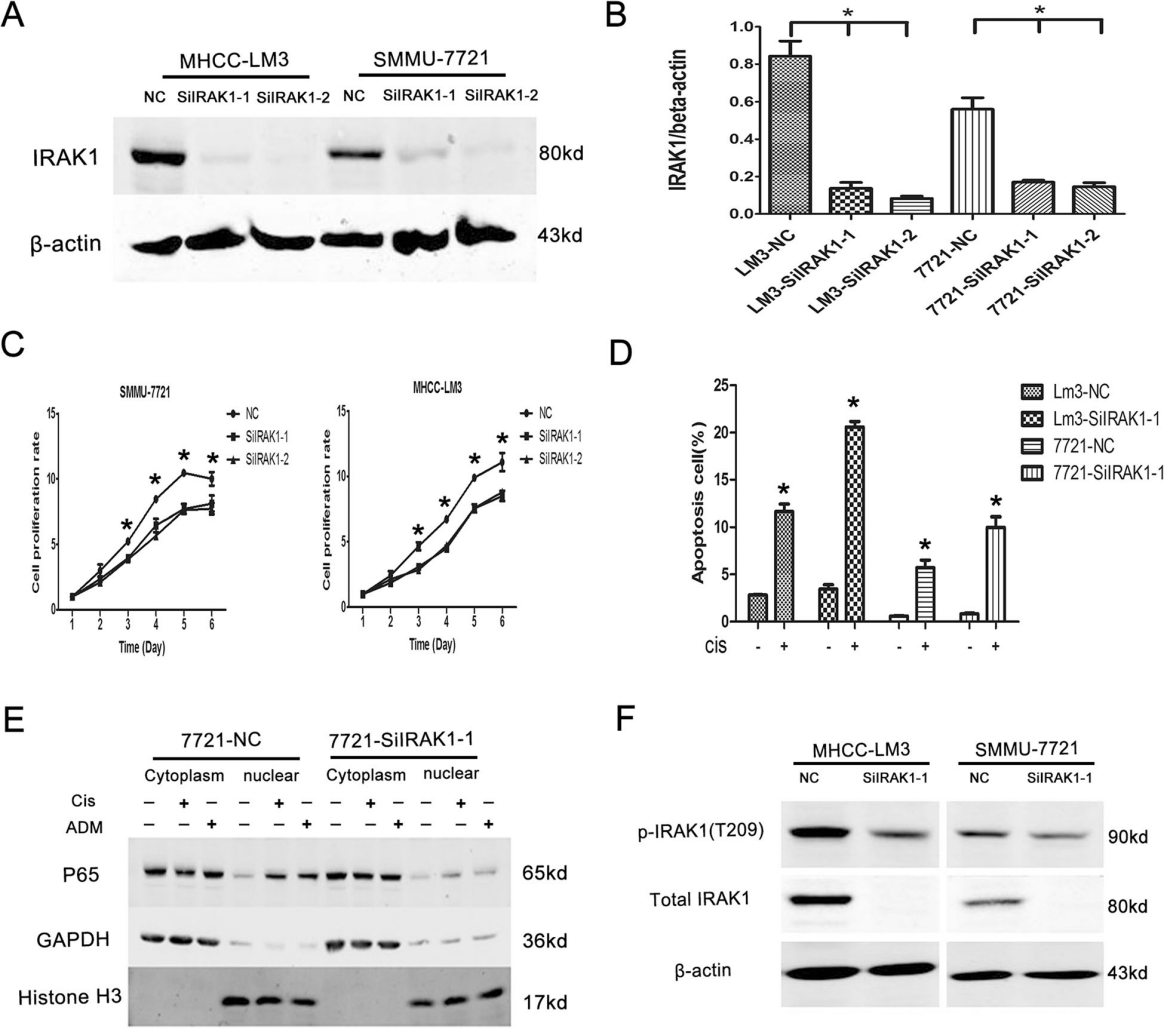
Knockdown of IRAK1 inhibited proliferation and induced apoptosis in HCC cells. **a** Western Blot analysis of IRAK1 in MHCC-LM3 and SMMU-7721 cell lines after IRAK1 knockdown. **b** The normalization expression of IRAK1/β-actin. **c** The proliferation curves of MHCC-LM3 and SMMU-7721 cell lines after IRAK1 knockdown. **d** Apoptosis analysis of MHCC-LM3 and SMMU-7721 cell lines with the stimulation of cisplatin (Cis, 25 μg/ml). **e** The nucleocytoplasmic separation analysis of SMMU-7721 cell lines under the stimulation of cisplatin (Cis, 25 μg/ml) or epirubicin (ADM, 5 μg/ml). **f** Western Blot analysis of p-IRAK1 (T209) after IRAK1 knocked down. NC: control cell lines; SiIRAK1-1: IRAK1 knockdown cell lines with s1 fragment sequence; SiIRAK1-2: IRAK1 knockdown cell lines with s2 fragment sequence; s1, s2: different shRNA fragments in si-IRAK1 lentivirus. * *P* < 0.05

To investigate the role of IRAK1 in HCC cell growth, the protein levels of IRAK1 in liver cancer cell lines were examined, including PLC/PRF/5, Huh7, HepG2, SMMU-7721 and MHCC-LM3. As shown in Fig. 2a and b, endogenous protein levels of IRAK1 were high in most cell lines. Considering the coherence of high expression of IRAK1 in HCC and most liver cancer cell lines, MHCC-LM3 and SMMU-7721 were interfered by lentivirus-mediated siRNA to stably suppress IRAK1 expression. Two different siRNAs were used to knockdown IRAK1 in both cell lines (Fig. 3a and b). Cells infected with control lentivirus grew rapidly, whereas cells infected with lenti-si-IRAK1 had inhibited the cell growth (Fig. 3c). At the same time, si-IRAK1 was able to augment the chemical drug cisplatin (Cis)-induced apoptosis in SMMC-7721 or MHCC-LM3 cell lines as shown in (Fig. 3d). Moreover, in the presence of cisplatin (Cis) or epirubicin (ADM), si-IRAK1 diminished the nuclear protein levels of NF-kB/p65 (Fig. 3e). Additionally, we detected the protein levels of p-IRAK1 (T209) in MHCC-LM3-SiIRAK1-1 and SMMU-7721-SiIRAK1-1 cell lines and found that the p-IRAK1(T209) levels were decreased as down-regulation of IRAK1 (Fig. 3f). Therefore, we assumed that down-regulation of IRAK1 slowed proliferation and induced apoptosis mainly through phospho-IRAK1 status [14, 16].

### Inhibition of p-IRAK1 impeded cell proliferation and migration

**Fig. 4:**
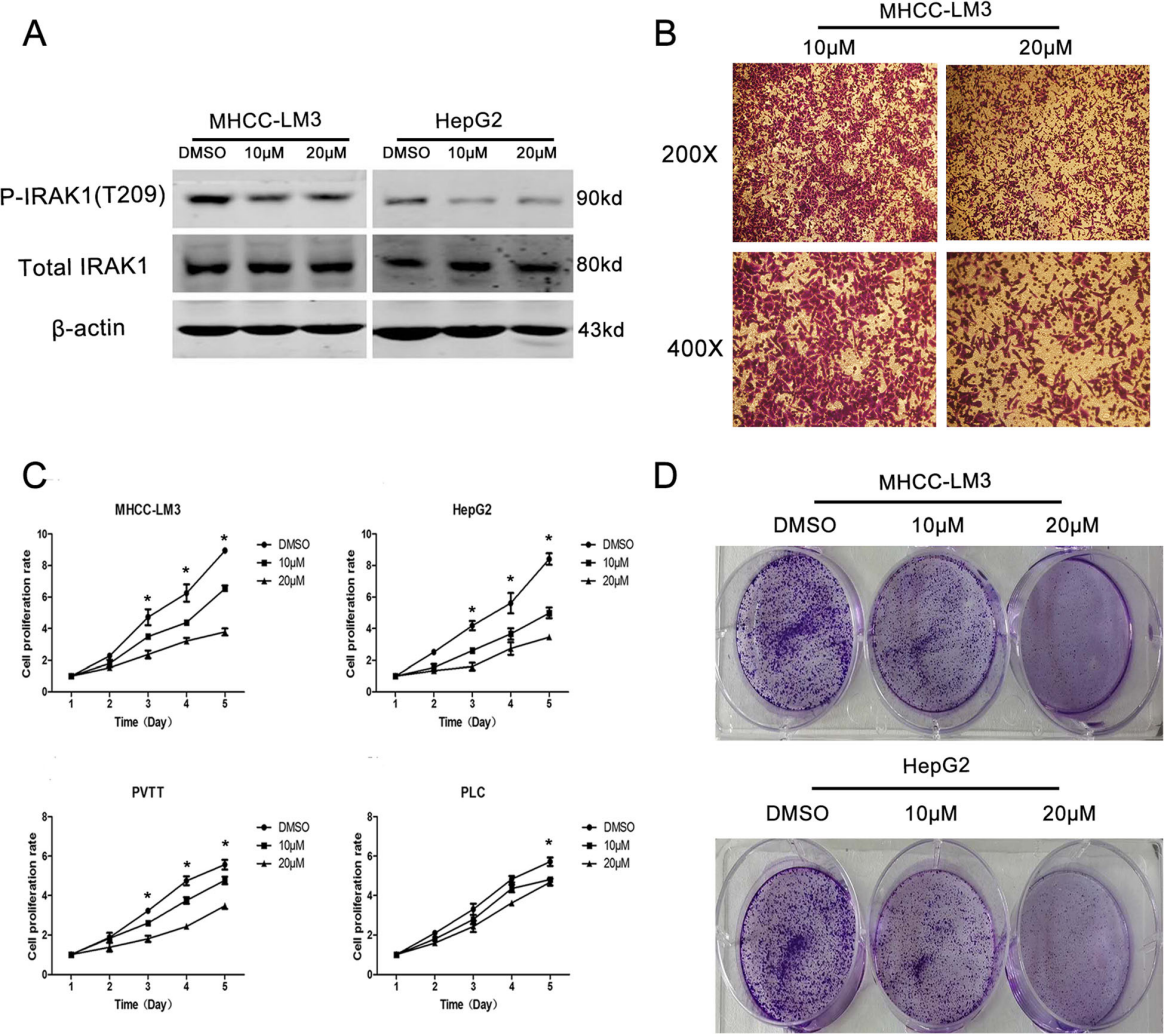
The inhibition of p-IRAK1 attenuated proliferation in HCC cells. **a** The protein levels of p-IRAK1 and total IRAK1 in SMMU-7721 and HepG2 with IRAK1/4 inhibitors (0, 10 μM, 20 μM). **b** Cell migration analysis in SMMU-7721 cell lines with IRAK1/4 inhibitors (0, 20 μM) treatment for 24 h. **c** The proliferation analysis in four different cell lines with IRAK1/4 inhibitors (0, 10 μM, 20 μM) for 1–5 days. **d** Colony formation analysis in SMMU-7721 and HepG2 cells with IRAK1/4 inhibitor (0, 10 μM and 20 μM) for 48 h

To further study whether p-IRAK1 (T209) contributes to HCC cells proliferation, the inhibitor of IRAK1/4, which selectively inhibits the activities of IRAK1 and IRAK4, was used to repress the activity of IRAK1 in SMMU-7721 and HepG2 cell lines. Sensitivity of IRAK1/4 inhibitor was evaluated by treating cells with serial dilutions of the drug for 5 days and then analyzing cell growth by CCK-8 assays. As displayed in Fig. 4a, phosphorylation of IRAK1 was significantly inhibited, which led to impaired proliferation of both cell lines in a dose-dependent manner. However, it was noted that the p-IRAK1 (S376), which might not be the main phosphorylated form in liver cancer cell lines, was almost not expressed in SMMU-7721 and HepG2 cell lines. After the inhibition of p-IRAK1 (T209) in four different cell lines, including SMMU-7721, HepG2, PVTT and PLC/PRF/5, the proliferation rates were greatly attenuated as showed in Fig. 4c, especially in the SMMU-7721 and HepG2. In addition, the colony formation analysis of SMMU-7721 and HepG2 cells with the IRAK1 inhibitor (0, 10 μM and 20 μM) for 48 h further confirmed the role of p-IRAK1(T209) in promoting HCC proliferation (Fig. 4d). It implicated that IRAK1 enhances proliferation in human HCCs mainly depending on its phosphorylation form (T209).

As is known, migration is crucial for HCC progression [32]. In this study, we further analyzed the effect of IRAK1 activity on HCC migration. As the results in Fig. 4b, the IRAK1/4 inhibitor (20 μM) decreased the number of migrated cells in SMMU-7721 cells, revealing that IRAK1 improves HCC migration.

### Inhibition of p-IRAK1 lessened cell cycle arrest but increased apoptosis in HCC cells

**Fig. 5:**
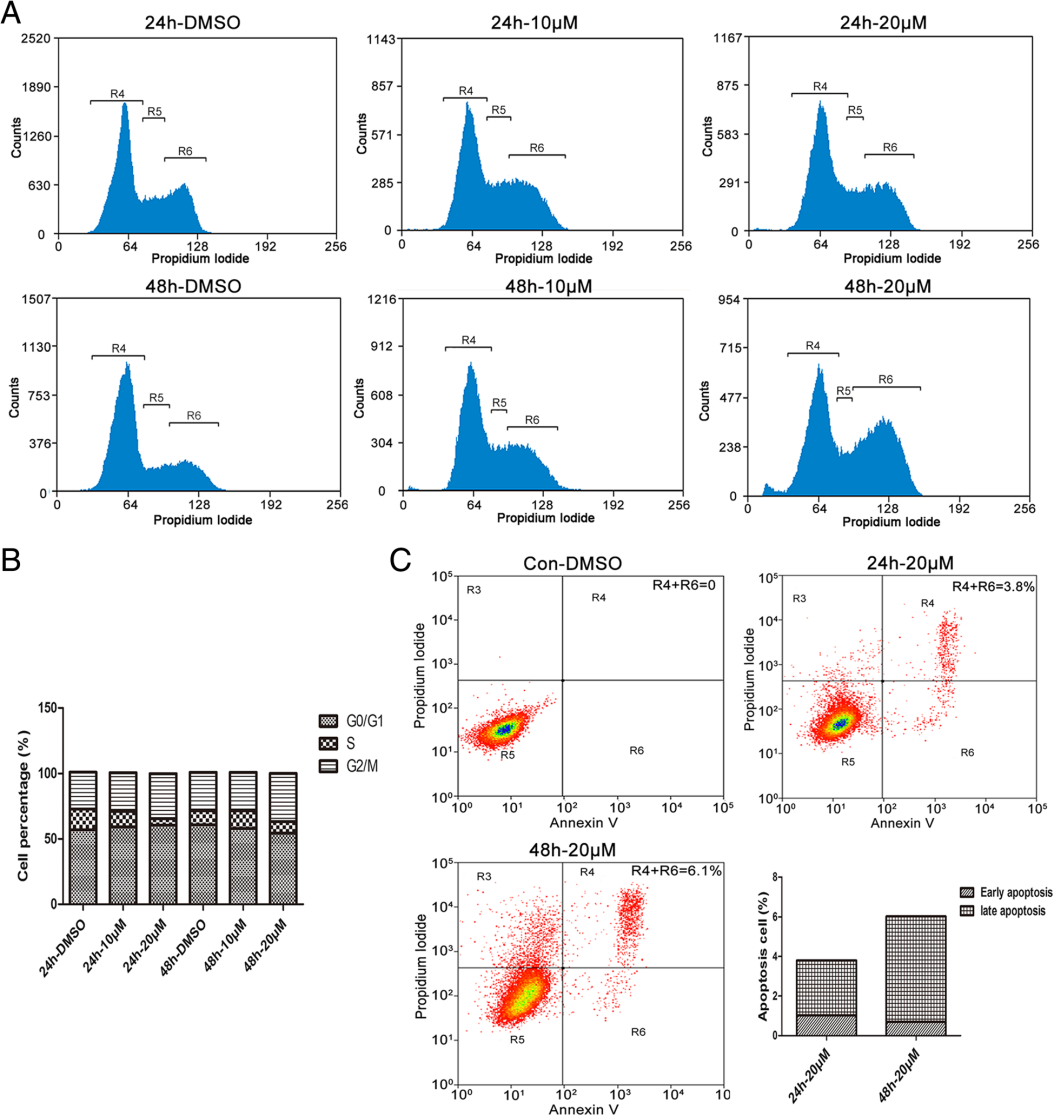
The IRAK1 inhibitor arrested the cell cylce and induced apoptosis in HCC cells. **a** Flow cytometry assays of cell cycle arrest in SMMU-7721 cell line, with treatment of IRAK1/4 inhibitors (0, 10 μM, 20 μM) for 24 h and 48 h and staining with PI. **b** The fractions of cells in each phase of the cell cycle including G0/G1, S and G2/M phases. **c** Flow cytometry assays of cell apoptosis in SMMU-7721 cell line, with treatment of the IRAK1/4 inhibitor (20 μM) for 24 h and 48 h and staining with Annexin V and PI

Generally, cell proliferation is closely corresponding with the regulation of cell cycle. However, whether the inhibition of p-IRAK1 affected the cell cycle was unknown. We further determined whether the inhibition of p-IRAK1 affected cell cycle distribution using flow cytometry. In Fig. 5a and b, IRAK1/4 inhibitor treatment (0, 10 or 20 μM) for 24 h or 48 h led to a decrease in the percentage of cells in S phase. Moreover, IRAK1/4 inhibitor treatment at the concentration of 20 μM also caused apoptosis at 24 h and 48 h in SMMU-7721 cells (Fig. 5c), suggesting the anti-apoptosis role of IRAK1 in liver cancer.

### The proliferation promoting of IRAK1 in subcutaneous tumor in vivo

**Fig. 6:**
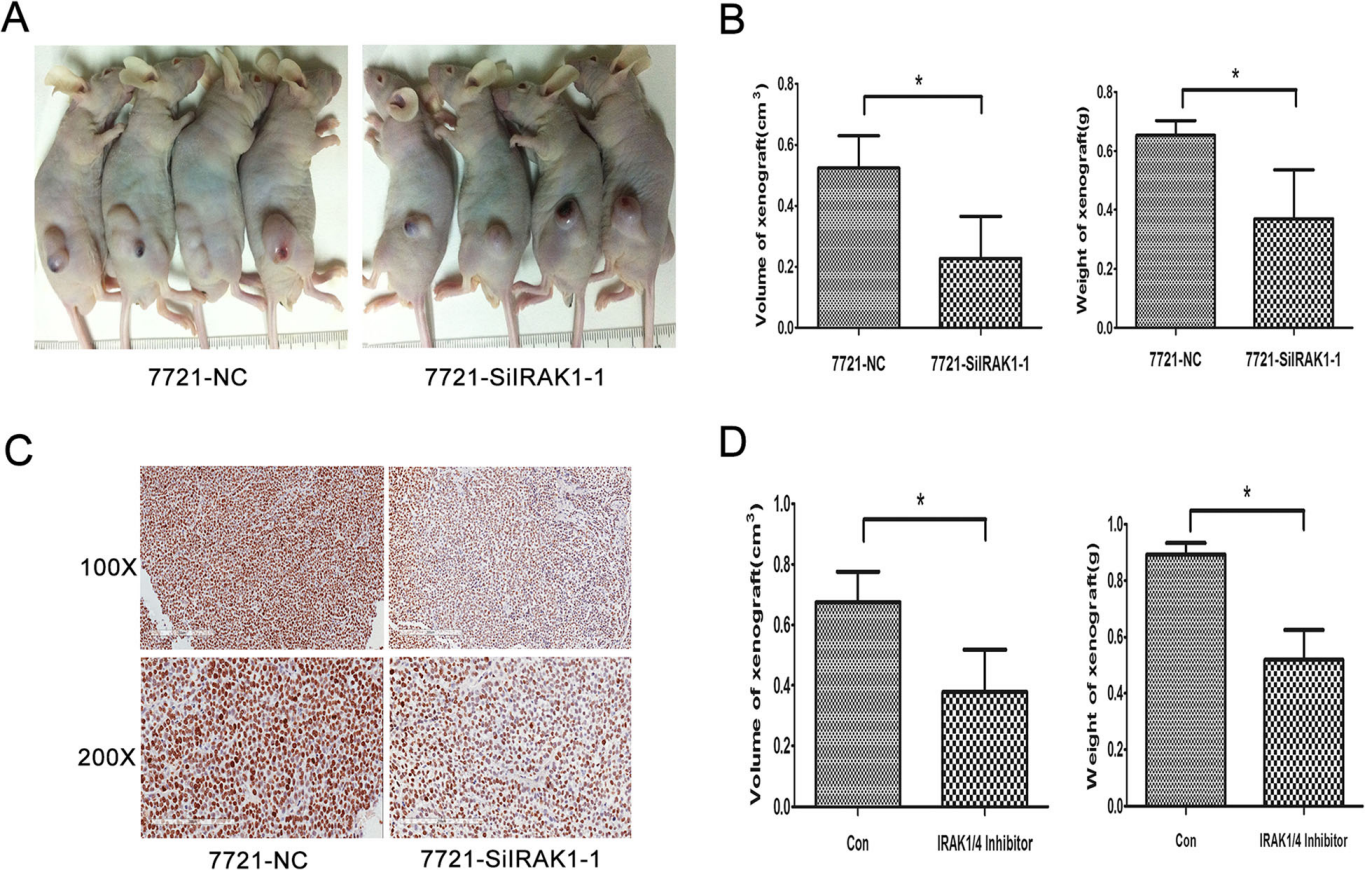
Inhibition of IRAK1 attenuates tumor growth in subcutaneous HCC models. **a** Xenografted tumor of SMMU-7721 cell lines stably transfected with lentivirus-delivering shRNA for IRAK1 (shIRAK2) in nude mice. **b** The graph shows changes in the volume or weight of tumors. The data are the mean ± SE (*n* = 4). **c** The Ki67 staining of Xenografted tumor (X100 and X200). **d** The graph shows changes in the volumes or weights of tumors. The data are the mean ± SE (*n* = 4). SMMU-7721 cell lines were implanted subcutaneously in mice and allowed to grow until the tumors reached a size of approximately 150 mm^3^. Xenografted mice were randomized and injected i.p. with 2.12 mg/kg IRAK1/4 inhibitor 4 times per week. Two weeks later, the average tumor volumes and weights of the IRAK1/4 inhibitor group were analyzed comparing with the control group. Tumor volumes from two groups were analyzed by two-way ANOVA analysis (*p < 0.05). The graph shows individual tumor volume (*t*-test, *p < 0.05)

To further study the promoting-tumor effects of IRAK1 in vivo, we employed SMMU-7721 HCC exnograft models. SMMU-7721-SiIRAK1-1 cells were injected subcutaneously into the flanks of nude mice. As presented in Fig. 6a and b, the average tumor volumes and weights in the SMMU-7721-SiIRAK1-1 groups were smaller than those in the NC control. Additionally, the immunohistochemical staining of Ki-67 also showed that shIRAK1 delayed SMMU-7721 cell proliferation in HCC xenograft model (Fig. 6c).

Moreover, based on SMMU-7721 HCC xenograft model, IRAK1/4 inhibitor treatment was also used to investigate the pro-tumor effects of IRAK1 in vivo. SMMU-7721 cells were injected subcutaneously into the flanks of nude mice. After the palpable xenograft tumors were established, the mice were randomly assigned into treatment with IRAK1/4 inhibitor or DMSO (control group). As shown in Fig. 6d, the average tumor volumes and weights in the IRAK1/4 inhibitor-treated groups were also smaller than those in the DMSO groups (*p* < 0.05). Mice treated with inhibitor showed no obvious signs of toxicity due to no difference among body weight, food and water intake, activity during treatment.

## Discussion

The pathogenesis of HCC is complex and diverse, involving different signal pathways, such as Wnt [33, 34], MAPK [30, 35, 36] and PI3K/AKT [31]. Recent studies also showed that inflammation signal pathways were closely related to tumorigenesis and development of HCC [37, 38]. As IRAK1 plays a key role in the TLRs/IL-1 signaling pathway by activating the downstream of NF-kB, the functions of IRAK1 in different tumors have been wildly focused. In acute myeloid leukemia (AML), over-expressed IRAK1 and universal activation were frequent [21]. In the melanoma cell lines, both IRAK4 and IRAK1 are highly expressed and activated, and promote primary melanoma progression [22]. IRAK1 has been proved as the therapeutic target for lung cancer [23, 24]. Moreover, a recent study showed over-expression of IRAK1 in breast cancer and demonstrated its potential target for triple-negative breast cancer (TNBC) metastasis to overcome paclitaxel resistance [26]. Christian Pilarsky et al. reported that *IRAK1* gene was over-expressed in 10 kinds of cancers, including liver cancer, but there was no further investigation of the function of IRAK1 [39].

Due to unrestrained proliferation is an important characteristic for most malignant tumors including HCC [40, 41], it is meaningful to study the related mechanism and seek a new therapy strategy. In this study, frequently high expressions of IRAK1 in HCC tissues and liver cancer cells were confirmed, revealing the crucial role of IRAK1 in HCC development. We focused on the effect of IRAK1 on cell proliferation, and found the promotive role of IRAK1 for cell proliferation by regulating cell cycle. Suppression of IRAK1, by either siRNAs or the pharmaceutical IRAK1/4 inhibitor, lessened cell proliferation in HCC cell lines in vitro and HCC xenograft tumor growth in vivo. A recent research of breast cancers [26] showed that over-expression of IRAK1 could promote TNBC growth through regulating NF-kB-related cytokines secretion. However, in liver cancer, our data were more prone to its regulation about S phase in cell cycle. Next, more efforts will be focused on the detail mechanism of IRAK1 in the cell proliferation in liver cancer.

Chemical inhibition of IRAK1 in melanoma cells resulted in increased apoptosis in vitro and in vivo [22]. Adam et al. [42] also discovered that genetic or pharmacologic inhibition of IRAK1 attenuated ERK1/2 pathway through TRAF6 and induced cell apoptosis in head and neck cancer cell lines. Combination increased apoptosis and reduced migration by IRAK1/4 inhibitor in HCC cell lines, IRAK1 is postulated to promote HCC progression by controlling HCC cell proliferation and apoptosis.

The pharmaceutical IRAK1/4 inhibitor has already been frequently used for acute myeloid leukemia (AML) treatments [21]. Our work further discovered that IRAK1/4 inhibitor as a novel strategy for HCC therapy.

The high expression (mRNA and protein) of IRAK1 as well as activated IRAK1 (T209) observed in myelodysplastic syndrome, acute myeloid leukaemia [19, 20], melanoma [22] and HCC [8] showed the probable correlation between IRAK1 and its phosphorylated activation. Since the function of IRAK1 mainly relies on its phosphorylated status in most tissues and cells [14, 16], in this study, si-IRAK1 or the IRAK1/4 inhibitor suppressed phosphor-IRAK1 protein levels, indicating that high expression of IRAK1 in HCC promotes cell proliferation and anti-apoptosis mainly through its phosphorylated status. Moreover, the data from xenograft of HCC cell line confirmed this possibility. Of course, it still remains further study on the molecular mechanism about this association observed in HCC.

## Conclusions

We revealed the important role of IRAK1 in promoting HCC growth and apoptosis, and discovered it as a candidate target for HCC treatment. More details about the mechanisms and personalized therapy need to be further studied in future.

## Abbreviations

HCC: Human hepatocellular carcinoma
IRAK1: Interleukin-1 receptor associated kinase 1
TLR: Toll-like receptor
